# Cooperative Regulation of *Campylobacter jejuni* Heat-Shock Genes by HspR and HrcA

**DOI:** 10.3390/microorganisms8081161

**Published:** 2020-07-30

**Authors:** Marta Palombo, Vincenzo Scarlato, Davide Roncarati

**Affiliations:** Department of Pharmacy and Biotechnology (FaBiT), University of Bologna, 40126 Bologna, Italy; marta.palombo2@unibo.it

**Keywords:** *Campylobacter jejuni*, heat-shock response, transcriptional regulation, HspR repressor, HrcA repressor, DNA/protein interaction

## Abstract

The heat-shock response is defined by the transient gene-expression program that leads to the rapid accumulation of heat-shock proteins. This evolutionary conserved response aims at the preservation of the intracellular environment and represents a crucial pathway during the establishment of host–pathogen interaction. In the food-borne pathogen *Campylobacter jejuni* two transcriptional repressors, named HspR and HrcA, are involved in the regulation of the major heat-shock genes. However, the molecular mechanism underpinning HspR and HrcA regulatory function has not been defined yet. In the present work, we assayed and mapped the HspR and HrcA interactions on heat-shock promoters by high-resolution DNase I footprintings, defining their regulatory circuit, which governs *C. jejuni* heat-shock response. We found that, while DNA-binding of HrcA covers a compact region enclosing a single inverted repeat similar to the so-called Controlling Inverted Repeat of Chaperone Expression (CIRCE) sequence, HspR interacts with multiple high- and low-affinity binding sites, which contain HspR Associated Inverted Repeat (HAIR)-like sequences. We also explored the DNA-binding properties of the two repressors competitively on their common targets and observed, for the first time, that HrcA and HspR can directly interact and their binding on co-regulated promoters occurs in a cooperative manner. This mutual cooperative mechanism of DNA binding could explain the synergic repressive effect of HspR and HrcA observed in vivo on co-regulated promoters. Peculiarities of the molecular mechanisms exerted by HspR and HrcA in *C. jejuni* are compared to the closely related bacterium *H. pylori* that uses homologues of the two regulators.

## 1. Introduction

All living organisms cope with thermal stress conditions by exploiting the activity of a class of highly conserved proteins known as Heat-Shock Proteins (HSP) [1]. These protective proteins can act as molecular chaperones, thereby mediating the proper folding of newly synthesised polypeptides or retrieving the functional structure of stress-denatured proteins. In addition, HSP include proteases, which are involved in the physiological turnover of cellular proteins and in the removal of amorphous protein aggregates, deleterious for cell viability [2,3]. Research efforts in recent decades have been focused not only on the definition of the HSP mechanisms of action, but also on the characterization of regulatory strategies adopted by different organisms to modulate HSP expression [4]. As a general rule, following a sudden temperature increase the synthesis of HSP is promptly stimulated by means of transcriptional and/or post transcriptional regulatory mechanisms. In bacteria, the heat-shock regulation at the transcriptional level has been shown to include highly diversified strategies, involving both positive and negative mechanisms. Positive regulation has been extensively studied in the model organism *Escherichia coli* and is based on the action of a heat-shock responsive sigma factor, which drives the RNA polymerase core enzyme on the promoters of heat-shock genes under stress conditions [4]. On the other hand, negative strategies of transcriptional regulation rely on dedicated repressors whose activity can be directly or indirectly influenced by environmental conditions. Prominent examples of heat-shock transcriptional repressors include HrcA, HspR and CtsR [1]. Specifically, under normal growth conditions the heat-shock genes’ transcription is repressed by the regulator occupying the core promoter region and interfering with RNA polymerase binding. In contrast, a sudden temperature increase provokes a direct or indirect reduction in the repressor affinity for its target operator, allowing the transcription to get started. Interestingly, in some bacterial species, positive and negative control mechanisms coexist and together regulate the expression of distinct sets of heat-shock genes [4].

*Campylobacter jejuni* is the leading cause of bacterial food-borne diarrhoeal disease worldwide [5]. While most cases of *C. jejuni*-associated gastroenteritis are generally self-limiting, some infections can evolve to severe complications, like the Gullian-Barré syndrome, a rare, autoimmune demyelinating disorder leading to muscle weakness and eventually paralysis [6]. Even though *C. jejuni* is a human pathogen, it is able to asymptomatically colonize the gastrointestinal tract of different animals, including several avian species. This ability provides the basis for the *C. jejuni* contamination cycle, in which contaminated water and raw milk or meat, especially poultry, serve as pathogen reservoirs, representing a source of human infection [7]. Considering the colonisation of different hosts (human and animals) and the ability to survive in the external environment, temperature could be a signal for triggering host-specific infection, and, in turn, the heat-shock response, a mechanism contributing to the switch between commensalism and pathogenicity [7,8]. *C. jejuni* has a small genome of 1.6 Mb with a limited number of genes coding for transcriptional regulators [9,10]. Its genome harbours homologues of *hrcA* and *hspR* while it lacks a dedicated heat-shock sigma factor, evoking a negative strategy of HSP regulation. Indeed, microarray-based transcriptomic analysis of *hrcA-* and *hspR-*mutant strains revealed that these regulators are directly or indirectly involved in the transcriptional control of several genes coding for all the major HSP [11,12]. Specifically, *hspR* inactivation led to the up-regulation of all the major heat-shock genes, including those coding for chaperones (the DnaK-DnaJ-GrpE system and the GroEL-GroES chaperonin) and proteases. In addition, a down-regulation of several flagellar-related genes has been observed, resembling the situation observed in the closely related human pathogen *Helicobacter pylori* [13,14]. The inactivation of *hrcA*, instead, provoked the up-regulation of three genes only, namely *groEL, groES* and *Cj0168c*, with the latter encoding a putative integral membrane protein [12]. The HspR repressor has been initially characterised in *Streptomyces* genus where it binds the HspR Associated Inverted Repeat (HAIR) sequence, which has the consensus CTTGAGT-N7-ACTCAAG [15,16,17]. Similarly, the HrcA regulator, originally studied in *Bacillus subtilis*, recognises a conserved operator sequence named CIRCE for Controlling Inverted Repeat of Chaperone Expression, which has the consensus TTAGCACTC-N9-GAGTGCTAA [18]. Interestingly, previous studies based on in silico approaches suggested the presence of inverted repeat sequences resembling HAIR and CIRCE mapping on the promoters controlling the expression of *C. jejuni* major HSP [11,12,19]. Nevertheless, an experimental demonstration of a direct regulatory role played by HrcA and HspR on heat-shock genes’ promoters is still missing.

In the present work, our primary aim was to characterise in vitro the mechanism of action of the regulators involved in the transcriptional control of heat-shock proteins’ expression in *C. jejuni.* To this end, we defined the binding sites of HrcA and HspR on the regulated promoters by performing DNase I footprinting experiments and showed that, under in vitro conditions, HrcA and HspR can directly interact with them and that their binding on co-regulated targets occurs in a cooperative manner. Furthermore, peculiarities of the molecular mechanisms exerted by HspR and HrcA in *C. jejuni* and in the closely related bacterium *H. pylori* are compared and discussed.

## 2. Materials and Methods

### 2.1. Bacterial Strains and Media

*E. coli* strain DH5α was used for cloning and plasmid preparations, BL21(DE3) strain was used for recombinant protein overexpression, and strain BTH101 was used for Bacterial Two Hybrid (BACTH, [20]) assays. *E. coli* strains were cultured in Luria–Bertani (LB) medium. When required, ampicillin and kanamycin were added to a final concentration of 100 and 25 μg/mL, respectively. *C. jejuni* NCTC11168 cells were recovered from frozen stocks on Brucella broth agar plates containing 5% fetal calf serum, under microaerophilic conditions at 37 °C and 95% humidity in a water jacketed incubator (Thermo Scientific, Waltham, MA, USA). Liquid cultures were grown in Brucella Broth supplemented with 5% fetal calf serum with gentle agitation (120 rpm), under microaerophilic conditions (Oxoid, Basingstoke, Hampshire, United Kingdom).

### 2.2. Molecular Biology Procedures

DNA manipulations were performed as described by Sambrook et al. [21]. All restriction and modification enzymes were used according to the manufacturer instructions (New England Biolabs, Ipswich, MA, USA). Mini- and midi-scale plasmid preparations were carried out with the Nucleospin plasmid and the NucleoBond Xtra Midi plasmid purification kits, respectively (Macherey-Nagel GmbH & Co., Düren, Germany). DNA fragments amplified through PCR for cloning purposes were purified from agarose gels with the NucleoSpin Gel and PCR Clean-up kit (Macherey-Nagel GmbH & Co, Dueren, Germany). For mutant promoter probes’ construction, the DNA fragments CjPcbpWT, CjPcbpMHL, CjPcbpMHR and CjPcbpMH were obtained by annealing the single-strand oligonucleotides CjPcbpWT-F and CjPcbpWT-R, CjPcbpMHL-F and CjPcbpMHL-R, CjPcbpMHR-F and CjPcbpMHR-R, and CjPcbpMH-F and CjPcbpMH-R (Table S1). In detail, the complementary DNA oligonucleotides were mixed in equal amounts and boiled for 10 min, then slowly chilled to room temperature. Finally, dsDNA probes were purified from agarose gel. Differently, the DNA fragments CjPgroWT, CjPgroMHL, CjPgroMHR, CjPgroMH, CjPgroMM, and CjPgroML were PCR amplified using the oligonucleotides CjPgroWT-F and CjPgroWT-R, CjPgroMHL-F and CjPgroMHL-R, CjPgroMHR-F and CjPgroMHR-R, CjPgroMH-F and CjPgroMH-R, CjPgroMM-F and CjPgroMM-R, and CjPgroML-F and CjPgroML-R (Table S1).

### 2.3. Overexpression and Purification of Recombinant Proteins

The protocols for recombinant His-tagged HrcA and HspR proteins’ overexpression and purification were adapted from previously described procedures used for *H. pylori* homologous regulators [22,23]. Specifically, *C. jejuni* His-tagged HrcA and HspR proteins were overexpressed in *E. coli* BL21 (DE3) cells transformed with the plasmids pET15b-*hrcA* and pET15b-*hspR* (Table S2), respectively. Overnight cultures were diluted 1:100 in fresh LB medium containing ampicillin and grown at 37 °C to an optical density at 600 nm (OD_600_) of 0.6. After lowering the temperature to 20 °C, protein expression was induced by the addition of 0.4 mM isopropyl-β-d-thiogalactopyranoside (IPTG) and carried out for 20 h at 20 °C. Bacterial cells were collected through centrifugation and stored at −80 °C. For His-HrcA purification, cells derived from a 500 mL culture were resuspended in 15 mL of Lysis Buffer A (50 mM NaH_2_PO_4_, pH 8.0; 300 mM NaCl; 10 mM imidazole; 10% glycerol; 5 mM TCEP; 0.5% Triton X-100) containing 1 mg/mL lysozyme and incubated for 1 h at 4 °C. Bacterial cells were further disrupted by six cycles of sonication. The soluble protein fraction was incubated with 500 μL of 50% Ni^2+^-NTA slurry (Qiagen, Inc., Hilden, Germany) for 1 h at 4 °C on a tilt-roll shaker. Samples were washed five times with 5 mL of Lysis Buffer A and four times with 5 mL of Wash Buffer A (50 mM NaH_2_PO_4_, pH 8.0; 300 mM NaCl; 20 mM imidazole; 10% glycerol; 5 mM TCEP). His-HrcA was eluted five times with one volume of Elution Buffer A (50 mM NaH_2_PO_4_, pH 8.0; 300 mM NaCl; 250 mM imidazole; 10% glycerol; 5 mM TCEP). A 3 kDa MWCO Centricon ultra-filtration unit (Merck-Millipore, Darmstadt, Germany) was used to concentrate the protein and to exchange the buffer to 10 mM Tris-HCl, pH 8.0; 300 mM NaCl; 10% glycerol; 5 mM TCEP; 0.05% NP-40-igepal. Purified His-HrcA protein was stored in aliquots at −80 °C. For His-HspR purification, bacterial cells derived from a 200 mL culture were resuspended in 10 mL of Lysis Buffer R (50 mM NaH_2_PO_4_, pH 7.5; 300 mM NaCl; 10 mM imidazole; 10% glycerol; 1.5 mM PMSF) containing 1 mg/mL lysozyme, incubated for 1 h at 4 °C and then sonicated. The soluble protein fraction was incubated with 500 μL of 50% Ni^2+^-NTA (Qiagen, Inc., Hilden, Germany) for 1 h at 4 °C on a tilt-roll shaker. Nonspecific proteins were removed by washing the slurry five times with Lysis Buffer R and then four times with Wash Buffer R (50 mM NaH_2_PO_4_, pH 7.5; 300 mM NaCl; 20 mM imidazole; 10% glycerol). His-HspR was collected by seven elution steps with one volume Elution Buffer R (50 mM NaH_2_PO_4_, pH 7.5; 300 mM NaCl; 250 mM imidazole; 10% glycerol). Finally, the recombinant protein was dialyzed against two changes of Footprinting Buffer (50 mM Tris-HCl, pH 8.0; 300 mM NaCl; 10% glycerol; 1 mM DTT; 0.05% NP-40-igepal) and stored in aliquots at −80 °C.

It is worth mentioning that we experienced several problems in purifying the recombinant His-HrcA protein. In fact, even though the expression of the protein was carried out at low temperature (20 °C), a significant fraction of the HrcA protein formed insoluble inclusion bodies. In addition, following protein purification, the stability of HrcA in solution was quite poor and the protein showed the tendency to aggregate, a behavior already observed for the homologous HrcA proteins of other organisms [23,24,25]. These drawbacks have not been an obstacle for DNA-binding assays, while they have been limiting issues for in vitro protein–protein interaction studies, in which higher amounts of proteins needs to be incubated in solution for longer times.

### 2.4. DNase I Footprinting

Linearized plasmids harboring the promoter regions of interest (listed in Table S2) were labelled as previously described [26]. Specifically, each plasmid was linearized with NdeI or NcoI digestion, 5′-dephosphorylated with Calf Intestinal Phosphatase and labelled with [γ^32^P]ATP (PerkinElmer, Waltham, MA, USA) and T4 polynucleotide kinase. Promoter DNA fragments were separated from the plasmid backbone with a second digestion with NcoI or NdeI and gel purified. Increasing concentrations of recombinant proteins were incubated with labelled probes for 15 min at room temperature in a 50 μL reaction containing 1× Footprinting Buffer (1× FPB: 5 mM Tris-HCl, pH 8.0; 60 mM NaCl; 5 mM KCl; 5 mM MgCl_2_; 0.2 mM DTT; 0.01% NP-40-igepal and 10% glycerol) and 200 ng of sonicated salmon sperm DNA as nonspecific competitor. Then, 0.066 units of DNase I, diluted in 1× FPB containing 5 mM CaCl_2_, were added to the mixture, and the reaction was incubated at room temperature for 75 s. The digestion was stopped by adding 140 μL of DNase I Stop Buffer (192 mM NaOAc, pH 5.2; 32 mM EDTA; 0.1% SDS; 64 μg/μL sonicated salmon sperm DNA). The digested DNA was phenol-chloroform extracted, ethanol precipitated and resuspended in 13 μL of Formamide Loading Buffer (95% formamide; 10 mM EDTA; 0.1% bromophenol blue). The samples were denatured for 5 min at 100 °C, chilled for 1 min on ice and subjected to 6% polyacrylamide-urea gel electrophoresis. The gel was blotted onto a 3 MM Whatman paper sheet (Thermo Scientific, Waltham, MA, USA), dried and autoradiographed.

### 2.5. GST-Pull-Down Assay

For the glutathione S-transferase (GST) pulldown assay, *C. jejuni* HspR and HrcA (baits) were overexpressed as a fusion protein with GST. In particular for the HrcA protein, the expression of the protein fused to GST, a large fusion tag known to enhance recombinant protein solubility and stability upon overexpression in *E. coli* cells [27], allowed us to partially solve the solubility/stability issues described above. As control bait, the GST protein alone was overexpressed and used in the pulldown assay under the same conditions as GST-HspR and GST-HrcA. The detailed protocol reported below was adapted from previously described procedures used for interaction studies on *H. pylori* homologous HspR regulator [28].

#### 2.5.1. Protein Overexpression and Slurry Interaction

*E. coli* BL21 (DE3) strain was transformed with the plasmids pGEX_NN_ or pGEX_NN_-*hrcA* and pGEX_NN_-*hspR* (Table S2) for the overexpression of GST or GST-tagged recombinant HrcA and HspR proteins, respectively. Overnight bacterial cultures were diluted 1:100 in fresh LB medium and grown to an OD_600_ of 0.6. Protein overexpression was induced by the addition of 1 mM IPTG for GST and GST-HspR or 0.4 mM IPTG for GST-HrcA and carried out for 20 h at 20 °C. The bacterial cells were collected, and the pellets were resuspended in 1× PBS, 1% Triton X-100, 1 mg/mL of lysozyme and incubated for 1.5 h at 4 °C before sonication. The soluble protein fraction was then incubated with 100 μL of Glutathione-Sepharose resin (GSH, Glutathione Sepharose^®^ 4B, GE Healthcare, Chicago, IL, USA) for 1 h at 4 °C. Samples were then washed four times with 1× PBS to remove nonspecific protein interactions and finally resuspended in 100 μL of 1× PBS. The bound GST-GSH Sepharose, GST-HspR-GSH Sepharose, and GST-HrcA-GSH Sepharose were quantified thought SDS-PAGE.

#### 2.5.2. GST Pull-Down and SDS-PAGE or Western Blot Analyses

Equimolar amounts of GST-GSH Sepharose, GST-HspR-GSH Sepharose or GST-HrcA-GSH Sepharose were incubated with 250 ng/μL of purified recombinant His-HspR in a final volume of 200 μL 1× FPB for 1.5 h at 4 °C. Samples were then washed seven times with 1× FPB and the pulled-down proteins analysed by SDS-PAGE and Western blot assay. For Western blot analysis, the proteins were separated by SDS-PAGE and blotted onto a nylon (PVDF) membrane (GVS North America, Sanford, Maine, USA) using a wet transfer apparatus (BioRad, Hercules, CA, USA). The transfer was conducted for 1 h at 150 V in Transfer Buffer (48 mM Tris, 39 mM glycine, 20% methanol, 0.037% SDS). Membranes were incubated in Blocking Buffer (5% low-fat milk in 1× PBS, 0.05% Tween-20) for 1 h at room temperature. Membranes were then rinsed three times for 5 min in 1× PBST (0.05% Tween-20) and incubated with the primary antibody (dilution 1:5000 of mouse anti-His clone HIS.H8 monoclonal antibody, Sigma-Aldrich, St Louis, MO, USA) in 5% low fat milk-1× PBST, for 1 h at room temperature. After three washing steps in 1× PBST, the membranes were incubated with a 1:10,000 dilution of a horseradish, peroxidase-conjugated, anti-mouse secondary antibody for 3 h at room temperature. Following three washing steps in 1× PBST, the immunoblot was developed by pouring on the membranes a solution of 1.25 mM luminol containing 0.015% H_2_O_2_ and 0.068 mM p-coumaric acid. The signal was visualized by film-autoradiography.

## 3. Results

### 3.1. The HspR Repressor Directly Binds to Extended DNA Regions Mapping within Several C. jejuni Heat-Shock Promoters

**Figure 1:**
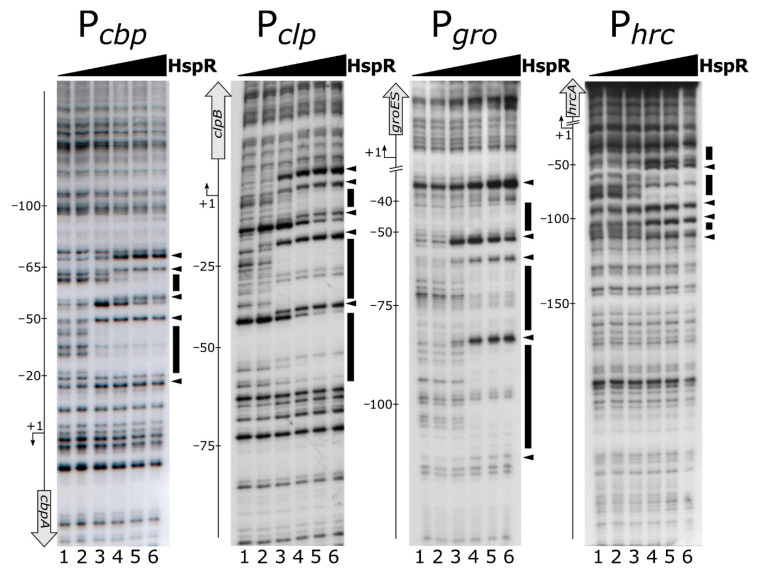
Mapping of HspR binding sites on heat-shock promoters. DNase I footprinting assays of HspR on P*cbp*, P*clp*, P*gro* and P*hrc* promoter probes (panels A, B, C and D, respectively). Radiolabelled DNA fragments were incubated with increasing concentrations of the purified HspR protein (0, 11, 22, 45, 90 and 180 nM HspR; lanes 1 to 6, respectively) and subjected to DNase I digestion. In each panel, on the right, black boxes highlight protected regions and black arrowheads indicate bands of hypersensitivity to DNase I digestion. On the left, the bent arrow indicates the transcriptional start site and the vertical open arrow depicts the indicated open reading frame; numbers refer to the positions with respect to the transcriptional start site (annotated according to [29]).

To assess if the HspR repressor directly binds to heat-shock promoter regions and, if this is the case, to precisely map its binding sites, a recombinant His-tagged HspR was expressed, purified and used in DNase I footprinting assays on the P*cbp*, P*clp*, P*gro* and P*hrc* promoters. Figure 1 shows for all the probes tested that the addition of increasing concentrations of recombinant HspR protein led to the appearance of DNase I hypersensitive sites (highlighted by black arrowheads) flanked by protected regions (evidenced by black boxes). A detailed analysis of the location and extension of HspR contacted regions highlighted that the repressor binds to extended DNA regions of different sizes and mapped in different positions on the assayed promoters with respect to the transcription start sites. In detail, as evident from Figure 1, the protected regions extended from position −20 to position −65 and from position +18 to position −63 with respect to the transcriptional start sites of the P*cbp* and P*clp* promoters, respectively, thereby overlapping the core promoter region. On the contrary, DNA binding experiments on the P*gro* and P*hrc* promoters showed that in these cases HspR protected more extended regions (from position −37 to position −106 for P*gro* and from position −16 to position −111 for P*hrc*), which are located upstream of the core promoter.

Intriguingly, we noticed that the protection pattern of HspR on the DNA probes showed a first protected region at low protein concentrations which then extends to other areas of protection at higher protein concentrations, suggesting the existence of high- and low-affinity binding sites. For example on P*cbp*, upon addition of 22 nM HspR, a region of protection and bands of enhanced DNase I sensitivity appeared between positions −20 and −55 (Figure 1, P*cbp*, lane 3). At higher concentrations of HspR, a second protected region and two additional hypersensitive sites close to position −65 (Figure 1, P*cbp*, lanes 4–6) were observed. Analogous results were obtained on P*clp*, P*gro* and P*hrc* promoter probes (Figure 1).

In summary, DNase I footprinting assays demonstrated that HspR directly binds to extended DNA regions mapping to different positions with respect to the transcriptional start sites on heat-shock promoters. In addition, all the promoters tested seem to include high- and low-affinity binding sites for the HspR repressor.

### 3.2. Sequence Analysis of HspR Binding Sites

**Figure 2:**
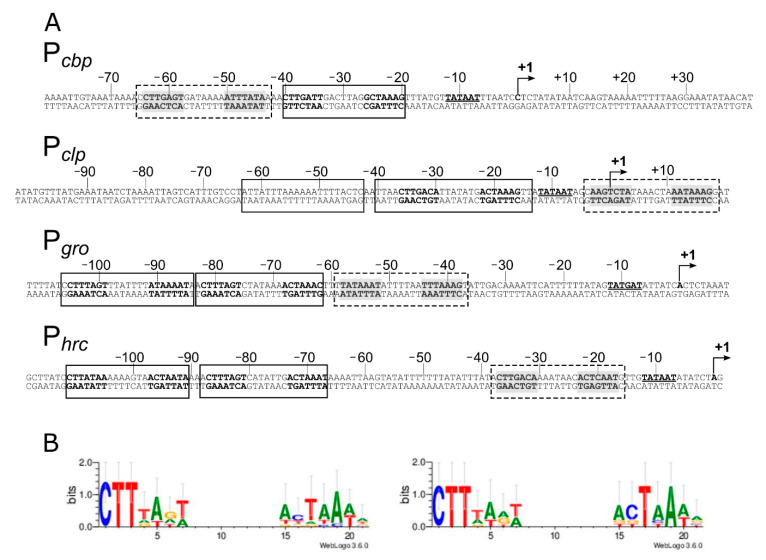
Features of the heat-shock promoter sequences and definition of HspR consensus binding motif. Panel **A**: for each promoter sequence, the numbers refer to the positions with respect to the transcriptional start site (+1), and the −10 promoter element is in boldface type underlined on the coding DNA strand. The HspR Associated Inverted Repeat (HAIR)-like sequences are represented in boldface on both strands. The HAIR-like sequences identified in this study are shaded in grey. The DNase I protected regions are enclosed in boxes: solid line boxes indicate high-affinity binding sites, while dashed line boxes delimit low-affinity binding regions. Panel **B**: HAIR consensus motifs derived in this study, visualized by inputting WebLogo with the alignment of all identified HAIR (left panel) or the alignment of the HAIR sequences bound with higher affinity by HspR (right panel). In both sequence logos, the height of each letter represents the relative conservation of each base.

In vitro DNA-binding assays described in the previous paragraph (Figure 1) indicate that the HspR repressor directly contacts extended regions characterized by protected areas alternating with hypersensitive sites to DNase I. It has been previously proposed that each heat-shock promoter included in our analysis (P*cbp*, P*clp*, P*gro* and P*hrc*) encompasses one HAIR-like sequence, resembling the inverted repeat (IR) consensus sequence (CTTGAGT-N7-ACTCAAG) originally defined in *Streptomyces coelicolor* as the HspR binding site [11,12,30]. Notably, nucleotide sequence analysis of the identified DNA regions bound by HspR revealed the existence of additional HAIR-like sequences not previously proposed. As reported in Figure 2A, we identified one additional sequence resembling the HAIR-motif on both the P*cbp* and the P*clp* promoters, while two new HAIR-like sequences were spotted both on the P*gro* and the P*hrc* promoters. These newly identified HAIR-like sequences are characterized by a lower degree of conservation than the already known HAIR with respect to the *S. coelicolor* consensus motif. In addition, as can be appreciated by comparing the positions of high- and low-affinity binding sites (Figure 1) and the positions of the HAIR-motifs on heat-shock promoters (Figure 2A), we noticed a correlation between HAIR sequence degree of conservation with respect to the *S. coelicolor* consensus motif and in vitro observed DNA-binding affinity of the HspR protein.

To characterize the consensus motif recognized by the HspR in *C. jejuni* and to identify key conserved nucleotides, HAIR-like motifs were aligned and used as input for the WebLogo software with results shown in Figure 2B. Alignment of all identified HAIR sequences revealed a conserved motif consisting of an imperfect IR of 21 bp (Figure 2B, left panel). The most conserved nucleotides (CTT) appear in the first three-nucleotide positions of the left hemisite. On the other hand, the right hemisite consists of an AT-rich sequence and displays a lower degree of conservation. By limiting the analysis to the HAIR sequences of the high-affinity HspR bindings, the degree of sequence conservation between the two arms of the inverted repeat increases (Figure 2B, right panel). This observation supports the hypothesis that differences between the high- and low-affinity HAIR lie in the sequence variations of the right arm of the inverted repeat, while the left arm of the inverted repeat is similarly conserved.

### 3.3. High-Affinity HAIR-Like Motifs are Necessary to Enhance HspR-DNA Interaction to Flanking Low-Affinity Sites

To study the functional importance of the two arms of the HAIR inverted repeat, we mutagenized one or both hemisites of the high-affinity HAIR IR and assayed their ability to interact in vitro with HspR by DNase I footprintings. Specifically, for this analysis we selected the P*gro* promoter, which exhibits a central high-affinity binding site surrounded by two low-affinity sites, mapping upstream of the core promoter region. Mutagenesis of the central high-affinity binding site was carried out by changing each base and maintaining the same base composition (Figure S4 and Table S1).

**Figure 3:**
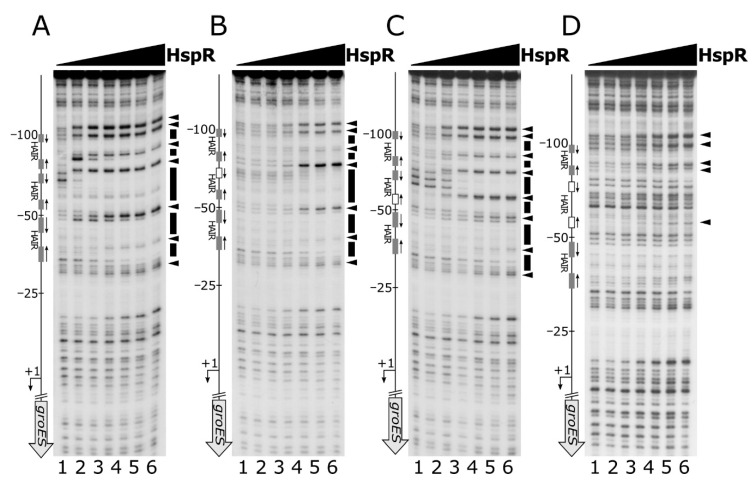
DNase I footprinting assays of HspR on wild type P*gro* (panel **A**) and on a set of mutant probes, comprising the mutant of the more conserved HAIR hemisite (panel **B**), of the less conserved HAIR hemisite (panel **C**) and the mutant of both HAIR hemisites (panel **D**). Radiolabelled DNA probes were incubated with increasing concentrations of recombinant HspR protein (0, 22, 45, 90, 180, 360 and 720 nM HspR; lanes 1 to 7, respectively) and subjected to partial DNase I digestion. On the right of each panel, black boxes depict the regions of protection and black arrowheads indicate bands of hypersensitivity to DNase I digestion. On the left side of each panel, a schematic representation of the promoter region, where the bent arrow indicates the transcriptional start site, while the vertical open arrow depicts the open reading frame and grey or white (if mutated) boxes alongside with converging arrows indicate the positions of the HAIR-like sequences; numbers refer to the positions with respect to the transcriptional start site.

Footprinting results showed that mutation of the more conserved HAIR-like hemisite led to a marked loss of HspR binding affinity, which is reflected in barely visible protected regions and faint hypersensitive bands, detectable only at high HspR concentrations (Figure 3, panel B compared to panel A). Base substitutions in the less conserved hemisite, instead, provoked a less pronounced effect on HspR binding affinity (Figure 3, panel C compared to panel A). Specifically, even though DNase I cleavage pattern slightly changed in this mutated DNA probe upon addition of increasing concentrations of protein (an additional DNase I hypersensitive band appeared around position −60), both regions of protection and DNase I hypersensitive sites in panel C became visible at lower HspR concentrations with respect to panel B. Finally, the mutation of both HAIR hemisites abolishes the HspR binding to the whole promoter region almost completely and allows the appearance of hypersensitive bands only at elevated HspR concentrations (Figure 3, panel D compared to panel A). Similar results were obtained on the P*cbp* promoter probe (Supplementary Figure S1), where the mutagenesis of one or both hemisites of the high-affinity HAIR-like induces an even more severe effect on HspR binding, if compared to the P*gro* promoter. All together, these results demonstrate that the interaction of HspR with DNA requires a full HAIR motif and that mutation of the high-affinity HAIR-like motif also severely impairs HspR binding to the flanking low-affinity binding site(s).

**Figure 4:**
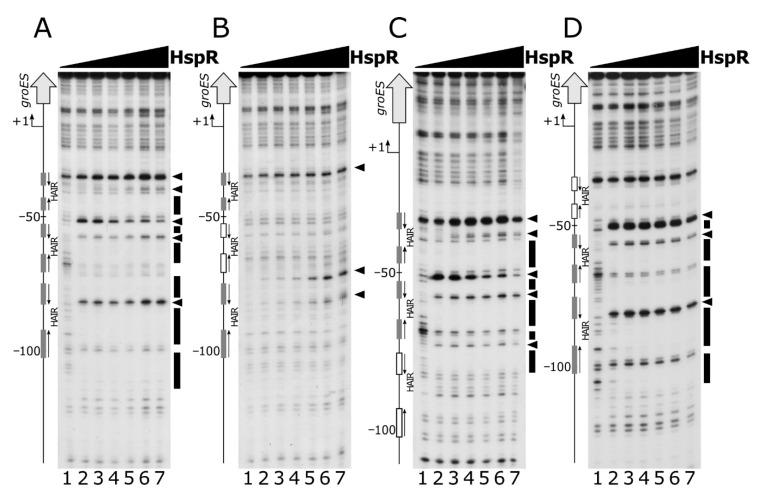
DNase I footprinting assays of HspR on wild type P*gro* (panel **A**) or on a set of mutant probes, comprising the mutant of the central high-affinity HAIR motif (panel **B**) and of the flanking low-affinity HAIR sequences (panel **C** and **D**). Reaction conditions and symbols are the same as in Figure 3.

Nevertheless, these data are not sufficient to understand the role played by the low-affinity HAIR-like sequences on heat-shock promoters. To shed light on the function of multiple HspR binding sites on heat-shock promoters, we carried out DNase I footprinting experiments on a panel of P*gro* mutant probes, in which the entire high- or low-affinity HAIR-motif were disrupted. For this panel of experiments, mutant probes were generated by changing each base and maintaining the same base composition (Figure S5 and Table S1). The DNase I footprinting results reported in Figure 4 indicated that disruption of the central high-affinity HAIR led to a dramatic decrease in the DNase I protection pattern by HspR (Figure 4, panel B compared to panel A). This observation reveals the crucial role of the central high-affinity HAIR for the recruitment of the HspR repressor on the promoter.

In contrast, footprinting experiments carried out on promoter probes mutated in the two low-affinity HAIR motifs revealed that loss HspR binding affinity was exclusively confined to the mutated region, while the protection pattern appeared unaltered on the adjacent binding sites (Figure 4, panel C and panel D). This result demonstrates that the low-affinity HAIR-like motifs are not necessary for HspR binding to high-affinity HAIR sequences.

In conclusion, all together these data support the pivotal role, at least on the promoters tested, of the high-affinity HAIR-like motif for HspR–DNA interaction, enhancing the repressor interaction with the flanking low-affinity sites. In addition, low-affinity HAIR-like sequences surrounding the high-affinity HAIR-like motif are necessary for HspR binding all over the promoter region.

### 3.4. The HrcA Repressor Directly Binds to CIRCE-Like Sequences Mapping within Pgro and Phrc Heat-Shock Promoters

**Figure 5:**
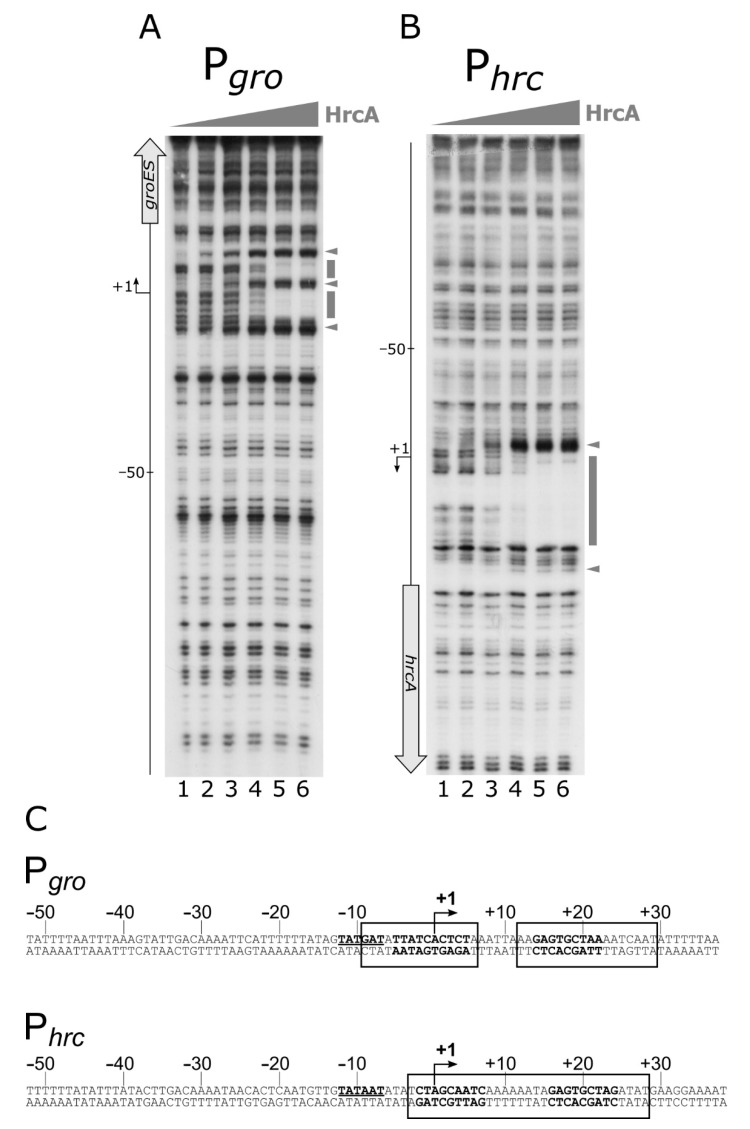
High-resolution mapping of HrcA binding sites on P*gro* and P*hrc* promoters. Panel **A** and **B**: DNase I footprintings of HrcA on P*gro* and P*hrc* promoters, respectively. The radiolabeled probes were incubated with increasing concentrations of recombinant HrcA protein (0, 22, 45, 90, 180 and 360 nM HrcA; lanes 1 to 6, respectively), followed by partial DNase I digestion. The symbols are the same as in Figure 3. Panel **C**: hallmarks of the P*gro* and the P*hrc* promoter sequences. In detail, numbers refer to the transcriptional start site (+1). The −10 box is underlined bold on the coding strands, and the CIRCE sequences are represented in boldface on both strands. Regions protected from DNase I digestion are enclosed in solid line open boxes.

Previous studies, in which transcriptome analyses were combined with in silico searches of IR sequences showing similarities to the CIRCE motif defined in *B. subtilis* as HrcA binding site, led to the identification of putative HrcA operators in both the P*gro* and P*hrc* promoter regions [12,19]. Surprisingly, the P*hrc* promoter was not found to be de-regulated in an *hrcA*-mutant background [12]. Starting from this information, we assayed HrcA direct interaction with both the P*gro* and the P*hrc* promoter regions through DNase I footprinting assays. Figure 5 clearly shows the ability of the HrcA repressor to bind and protect defined portions of both P*gro* and P*hrc* probes.

Specifically, the DNase I footprinting assay on P*gro* (Figure 5, panel A) showed three DNase I hypersensitive bands, which appeared starting from lanes 2 and 3, and two protected regions encompassing the transcriptional start site (+1 position). Interestingly, the same in vitro binding assay carried out on P*hrc*, showed that, following the addition of HrcA to the labelled probe, a clear protected area appeared, flanked on both sides by DNase I hypersensitive bands (Figure 5 panel B). In this case, the HrcA protected region encompasses the transcription start site. Figure 5, panel C, reports the sequences of the DNA regions protected from DNase I digestion by HrcA (enclosed in open boxes) on the two promoters. The HrcA binding site on both P*gro* and P*hrc* maps to a region encompassing the CIRCE motif previously proposed [12,19].

In conclusion, our data indicate that HrcA directly binds to both the P*gro* and P*hrc* promoters by contacting DNA regions overlapping the transcription start sites, and showing sequences similar to the CIRCE element, previously defined in *B. subtilis* as being the HrcA specific operator. In addition, it is interesting to highlight the fact that the HspR repressor binds upstream of the P*gro* and P*hrc* promoter elements, while the HrcA regulator binds to regions overlapping the core promoter and the transcription start site.

### 3.5. Cooperative Interaction of HspR and HrcA on the Co-Regulated Pgro Promoter

By comparing HspR and HrcA footprinting data presented above (Figure 1 and Figure 5), it appears that on P*gro* and P*hrc* co-regulated promoters, the distances between the HspR and HrcA binding sites are 27 and 11 bp, respectively. This is compatible with the hypothesis of a direct protein–protein interaction and a potential cooperative interaction of the two regulators on these common targets.

**Figure 6:**
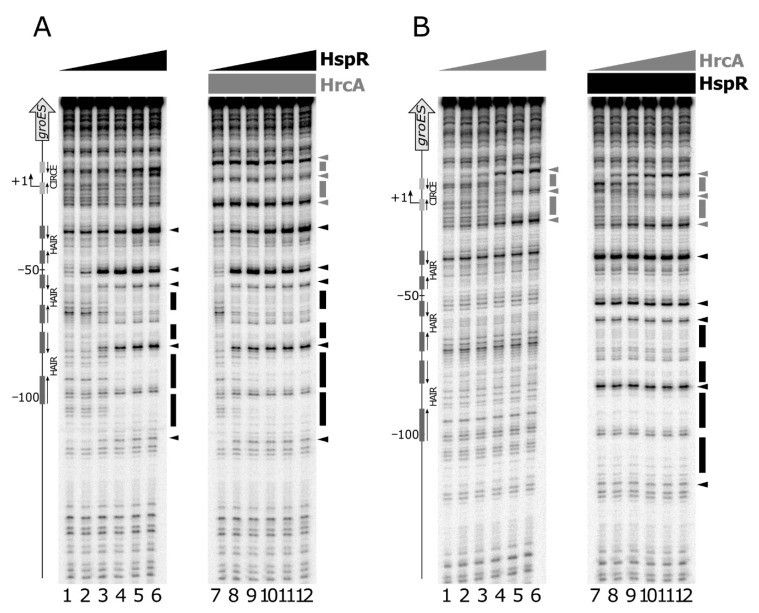
Panel **A**: DNase I footprinting analysis of HspR on the P*gro* promoter in the absence (left panel) and in the presence (right panel) of purified recombinant HrcA protein. A specific end-labeled P*gro* fragment was incubated with increasing amounts of purified HspR. Lanes 1 to 6: 0, 22, 45, 90, 180 and 360 nM HspR, respectively (in each reaction 360 nM GST was added as a control protein); lanes 7 to 12: 0, 22, 45, 90, 180 and 360 nM HspR, respectively (in each reaction, 360 nM HrcA was added). Molar ratio between HspR and HrcA, lanes 8 to 12: 1:16, 1:8, 1:4, 1:2 and 1:1. Panel **B**: DNase I footprinting analysis of HrcA on the P*gro* promoter in the absence (left panel) and in the presence (right panel) of purified recombinant HspR protein. A specific end-labeled P*gro* fragment was incubated with increasing amounts of purified HrcA. Lanes 1 to 6: 0, 22, 45, 90, 180 and 360 nM HrcA, respectively (in each reaction 360 nM GST was added as a control protein); lanes 7 to 12: 0, 22, 45, 90, 180 and 360 nM HspR, respectively (in each reaction 360 nM HspR was added). Molar ratio between HrcA and HspR, lanes 8 to 12: 1:16, 1:8, 1:4, 1:2 and 1:1. In each panel, regions protected from DNase I digestion are indicated by black (for HspR) or grey (for HrcA) boxes and the DNase I hypersensitive sites are depicted by black (for HspR) or grey (for HrcA) arrowheads. On the left, the bent arrow indicates the transcriptional start site, the vertical open arrow depicts the open reading frame; numbers refer to the positions with respect to the transcriptional start site. The HAIR and CIRCE sequences are indicated by dark and light grey boxes, respectively.

To study possible interactions between the two regulators, we assayed their DNA binding activities by performing DNase I footprinting assays on P*gro* under competitive conditions (Figure 6).

Panel A shows the results of DNA-binding assays carried out using HspR alone (lanes 1 to 6) and in combination with HrcA (lanes 7 to 12). The addition of increasing concentrations of HspR following HrcA binding to the probe led to an enhanced footprinting pattern compared to that obtained by incubating HspR in the absence of HrcA. This effect is clearly evident by comparing the intensities of all the sites of DNase I hypersensitivity, which emerge at lower HspR concentrations in the presence of HrcA. A comparable result was observed when increasing amounts of HrcA were added to HspR already bound to the DNA probe (Figure 6, panel B). In this case, the HrcA footprinting pattern is enhanced by the inclusion of HspR in the HrcA binding reactions (lanes 7 to 12), if compared to the control set (lanes 1 to 6). Therefore, we conclude that on the P*gro* promoter and under the in vitro conditions used, the two heat-shock repressors exhibit a mutual cooperative mechanism of DNA binding.

### 3.6. HrcA Directly Interacts with HspR

The DNA binding assays described above depict a novel scenario of HspR and HrcA binding architecture to heat-shock promoters, in particular, a direct mutual cooperative effect of HspR–HrcA interaction on co-regulated promoters. To assess this hypothesis, we performed a GST pull-down assay by incubating the purified recombinant GST-HspR and GST-HrcA fusion proteins with the recombinant His-tagged HspR (His-HspR). As a control, the same quantity of His-HspR was incubated with a recombinant GST protein. Following the incubation with a glutathione-sepharose slurry, the GST, GST-HrcA and GST-HspR proteins were recovered and the protein composition of the three samples was analysed through SDS-PAGE.

**Figure 7:**
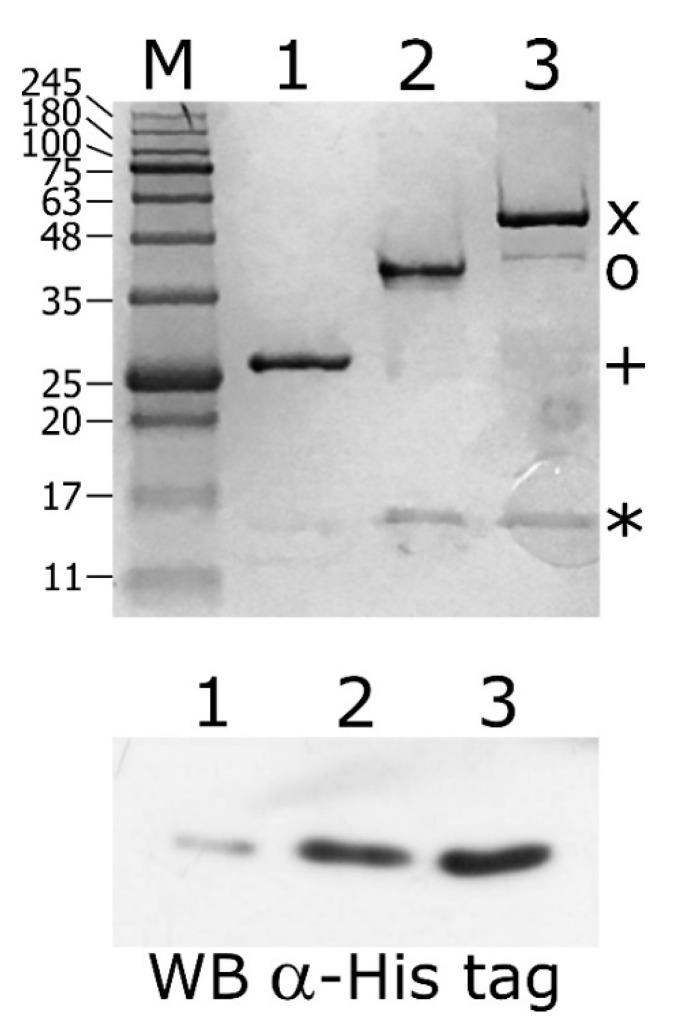
Glutathion S-transferase (GST)-pull down assay of purified His-HspR incubated with GST-HrcA or GST-HspR bound to Glutathione-Sepharose slurry. Upper panel: samples collected from the column containing GST alone (lane 1), GST-HspR- (lane 2) and GST-HrcA-GSH-Sepharose slurry (lane 3) were separated by SDS-PAGE together with molecular mass ladder (lane M) and stained with Coomassie Brilliant Blue. The bands corresponding to GST (+), GST-HspR (o), GST-HrcA (x) and His-HspR (*) are indicated on the right. Lower panel: samples collected from the column containing GST alone (lane 1) and GST-HrcA- (lane 2) and GST-HspR-GSH-Sepharose slurry (lane 3) were separated by SDS-PAGE, blotted on a nylon membrane and stained with anti-His Tag antibody.

When we used the GST-HspR fusion protein as bait, a protein band corresponding to the expected molecular weight of His-HspR appeared (Figure 7, upper panel, lane 2). The band corresponding to HspR was undetectable in the control sample in which the GST bait was used (Figure 7, upper panel, lane 1). Interestingly, when the GST-HrcA fusion protein was used as bait, we observed a protein band likely corresponding to the HspR prey (Figure 7, upper panel, lane 3). These samples were also analyzed by immunoblot stained with an anti-His tag antibody to recognize the His-tagged HspR protein (Figure 7, lower panel). These results strongly support the hypothesis of the tendency of HspR to form homodimers, but also a direct protein–protein interaction between HrcA and HspR. Similar indications were obtained by assessing the HspR and HrcA ability to form homo- and hetero-complexes through completely different techniques. Specifically, in vitro chemical crosslinking assays as well as bacterial two hybrid screenings confirmed that both HspR and HrcA can exist as homodimers and that the two heat-shock repressors can form hetero-complexes (Supplementary Figures S2 and S3).

## 4. Discussion

Bacterial cells respond to environmental stress insults by triggering a prompt accumulation of several proteins, including molecular chaperones and proteases. This cellular response guarantees protection against protein folding that is damaged by the stress insults. While some bacterial species, including the model organism *E. coli*, positively control the expression of heat-shock proteins through dedicated sigma factors, several regulatory strategies rely on the action of heat-shock transcriptional repressors. In these latter negative systems, the transcription of chaperones’ genes is maintained repressed during normal growth conditions by the repressors, while stress insults provoke the functional inactivation of the regulators, leading to derepression of target promoters. It has been previously shown that the major chaperone-encoding operons of *C. jejuni* are transcriptionally repressed by HspR, the homologue of the repressor of the *dnaK* operon of *Streptomyces* species. In addition, the transcription of two of the HspR-regulated operons, *groESL* and *hrcA-grpE-dnaK*, is also dependent on HrcA, the homologue of the repressor of the *groESL* operon of *B. subtilis* [11,12]. The presence of both regulators is therefore necessary for maintaining P*gro* and P*hrc* in the repressed state.

**Figure 8:**
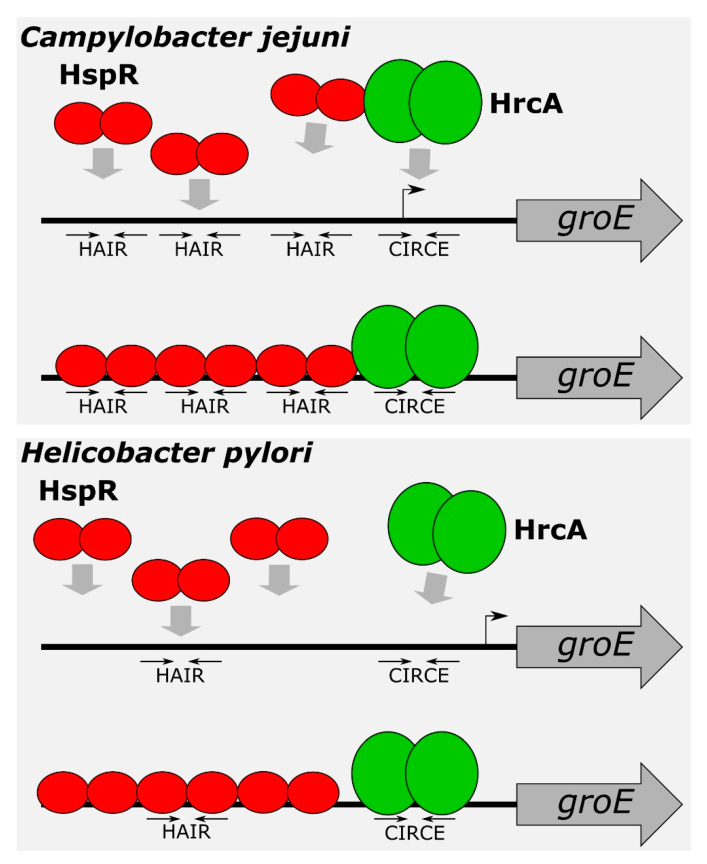
Model for P*gro* co-regulation by HspR and HrcA in *C. jejuni* (upper panel) and *H. pylori* (lower panel). In *C. jejuni*, HspR binds to multiple HAIR sequences located upstream the core promoter, covering an extended DNA region. High-affinity central HAIR inverted repeat is crucial for HspR-promoter interaction, which enhances the repressor interaction with the flanking low-affinity sites. HrcA binds to CIRCE-like motif located on the transcription start site. In *C. jejuni*, HspR and HrcA exhibit a mutual cooperative mechanism of DNA binding and a direct protein–protein interaction. In *H. pylori*, a dimer of HspR binds with high affinity to a single HAIR consensus sequence. This first binding event could act as a nucleation center, promoting cooperative binding of two HspR dimers on the flanking regions, likely recognizing still uncharacterized sequence determinants [14]. HrcA binds to a CIRCE element located in the core promoter. However, in *H. pylori,* HspR and HrcA bind to their operators in an independent manner and do not interact.

In the present study, we demonstrated the direct role played by both repressors on several heat-shock promoters and defined, at the molecular level, the DNA-binding architecture of both HrcA and HspR, contributing to the characterization of the regulatory circuit controlling the expression of the operons containing the major *C. jejuni* chaperones’ genes. The characterisation of HspR interactions with heat-shock promoters allowed us to define an interesting scenario. The HspR repressor binds to regulated promoters by contacting extended DNA regions, which include multiple binding sites, each one displaying a variable propensity to interact with the repressor (Figure 1). Sequence analysis of the DNA-protected regions evidenced that all HspR binding sites show an inverted repeat with similarities to the HAIR consensus sequence of *Streptomyces* spp. [30]. Notably, we noticed a correlation between HAIR sequence degree of conservation with respect to the *S. coelicolor* consensus motif and in vitro observed affinity of the HspR protein (Figure 1 and Figure 2A). This promoter layout is similar to what has been observed in *S. coelicolor*, where HspR binds extended DNA regions harboring three inverted repeat sequences in the promoter region of its DNA targets [15,16,30,31]. On the other hand, this is in striking contrast with the situation observed in *H. pylori,* in which each binding region harbours only one HAIR inverted repeat, even though HspR binding extends outside of the conserved sequence motif [14] (Figure 8). Alignment of high- and low-affinity HAIR sequences (Figure 2B), combined with DNase I footprinting experiments on mutated P*gro* and P*cbp* promoter sequences (Figure 3, Figure 4 and Figure S1) elucidated the pivotal role of the high-affinity HAIR-like motif for HspR-DNA interaction, enhancing the repressor interaction with the flanking low-affinity sites. In addition, our results show that low-affinity HAIR-like sequences surrounding the high-affinity HAIR-like motif are necessary for HspR binding all over the promoter region (Figure 4). This evidence allows proposing a DNA-binding mechanism where a first HspR-promoter interaction on the high-affinity site could act as core element that cooperatively stimulates the interaction of other HspR functional units on the flanking low-affinity binding sites. These sequential binding events might stabilise the HspR interactions with target promoters and strengthen the formation of the repressor–promoter complex (Figure 8, upper panel). In addition, data presented in this study allow speculation on the role of multiple binding sites with different affinities for the HspR repressor in a cellular environment. Possibly, they contribute to modulation of the concentration of functional HspR on promoters, and in turn, to the fine regulation of the target genes’ expression.

Even though HspR alone is sufficient to repress P*clpB* and P*cbp* promoters, both HspR and HrcA are needed to repress P*gro* and P*hrc* transcription under normal conditions of growth [12]. Precise mapping of HspR operators on all these heat-shock promoters (Figure 1 and Figure 2A) evidenced that, whereas on the P*cbp* and P*clp* promoters (controlled exclusively by HspR) HspR binding occurs in a region overlapping the core promoter elements, the HspR binding sites on the P*gro* and P*hrc* promoters map far upstream of the core promoter region and the transcriptional start site, in a non-canonical position for a transcriptional repressor (Figure 1 and Figure 2A). On HspR-HrcA co-regulated promoters, the core promoter regions, led unoccupied by HspR, are bound by its partner HrcA: this repressor covers a compact DNA region of about 30–40 bp, which encloses the transcription start site and includes the CIRCE-related inverted repeats (Figure 5 and Figure 8). It is likely that the binding of HrcA to these DNA elements represses transcription by steric interference of RNA polymerase binding. These observations are consistent with the typical architecture for dually regulated promoters observed in some other bacterial species [4], and support the role of HrcA as an HspR co-repressor also in *C. jejuni*.

A major finding of the present work concerns the mutual cooperative mechanism of DNA binding exhibited by HspR and HrcA on the co-repressed P*gro* promoter (Figure 6). On this promoter, the two repressors’ binding sites are very close to each other and both regulators appear to be necessary for full repression (Figure 1, Figure 2A and Figure 5A,C) [12]. In addition, we observed in vitro a reciprocal stimulation of their DNA binding activity (Figure 6) and a direct protein–protein interaction (Figure 7, Figures S2 and S3). Recalling the functional co-dependence of HspR and HrcA on co-regulated targets, our data suggest the possibility that this interaction could influence their recruitment in vivo on P*gro* and P*hrc* promoters and, in turn, contribute to heat-shock regulation of GroE and DnaK expression. In other words, HspR-HrcA protein–protein interaction may represent a prerequisite for the formation of a stable repression-competent complex on co-regulated target promoters. To the best of our knowledge, this is the first demonstration of a direct functional interaction between the two widespread heat-shock repressors HrcA and HspR. It is worth mentioning that in several other bacterial species, the regulons of different heat-shock repressors partially overlap, resulting in some genes being simultaneously controlled by two regulatory proteins. For example, the *groESL* promoter transcription is controlled by the concerted action of HrcA and CtsR in *Streptococcus pneumoniae* and *Staphylococcus aureus* [32,33], and the *acr2* and *groEL2* genes in *Mycobacterium tuberculosis* are regulated by the interplay among PhoP, HrcA and HspR [34,35]. In this latter example, the virulence-associated protein PhoP acts as a nodal regulator, able to alternatively interact with and recruit in vivo the HrcA and HspR repressors within the target genes and regulate the stress-specific expression of heat-shock proteins. Moreover, in this case, the independent functioning of HspR and HrcA heat-shock repressors has been demonstrated. In the human pathogen *H. pylori*, chaperones genes’ regulation by HspR and HrcA is built on an almost identical circuitry to the one here drawn for *C. jejuni*, where the regulon of HrcA appears to be entirely enclosed within the HspR regulon [36]. However, in *H. pylori* the two regulatory proteins bind to their operators in an independent manner and do not display any direct protein–protein interaction [13,37]. Indeed, in a previous work by our group, we assayed the DNA binding activities of HspR and HrcA under the same competitive conditions used in this work for the homologous regulators of *C. jejuni*. The results we got indicated that the two regulatory proteins of *H. pylori* bind to their operators in an independent manner [13]. Figure 8 provides a comparison between the proposed mechanisms of P*gro* dual regulation by HspR and HrcA in *C. jejuni* and *H. pylori*.

Together, the results reported in this work provide new mechanistic insights into the complex regulation of heat-shock responsive genes of *C. jejuni* and describe a novel example of complex heat-shock genes’ regulation characterised by the cooperative action of HspR and HrcA repressors.

## Supplementary Materials

The following are available online at https://www.mdpi.com/2076-2607/8/8/1161/s1, Figure S1: DNase I footprinting assays of HspR on wild type and mutant P*cbp* probes, Figure S2: In vitro crosslinking assay of HrcA and HspR proteins with formaldehyde, Figure S3: Bacterial Adenylate Cyclase Two-Hybrid System (BACTH) assays, Figure S4: Schematic representation of wild type and three different P*gro* mutant probes used in footprinting experiments reported in Figure 3, Figure S5: Schematic representation of wild type and three different P*gro* mutant probes used in footprinting experiments reported in Figure 4, Table S1: List of oligonucleotides used in this study, Table S2: List of plasmids used in this study.
