# Supplementary material for: Cooperative Regulation of Campylobacter jejuni Heat-Shock Genes by HspR and HrcA
Source: Microorganisms. 2020 Jul 30;8(8):1161. doi: 10.3390/microorganisms8081161 (PMC7464140; doi:10.3390/microorganisms8081161)
Supplement: Supplementary file 1 [file microorganisms-08-01161-s001.pdf]

Supplementary Materials

Cooperative regulation of *Campylobacter jejuni* heat-shock genes by HspR and HrcA

Marta Palombo, Vincenzo Scarlato* and Davide Roncarati*

Table of contents
Pag.

Figure S1: DNase I footprinting assays of HspR on wild type and mutant P*cbp* probes...……………….…….. 2

Figure S2: In vitro crosslinking assay of HrcA and HspR proteins with formaldehyde.…….…...……………. 3

Figure S3: Bacterial Adenylate Cyclase Two-Hybrid System (BACTH) assays……………………..…………..4

Figure S4: Schematic representation of wild type and three different P*gro* mutant probes used in footprinting experiments reported in Figure 3……………………………………………………………………………..…5

Figure S5: Schematic representation of wild type and three different P*gro* mutant probes used in footprinting experiments reported in Figure 4……………………………………………………………………………………6

Table S1: List of oligonucleotides used in this study………………………………………………………………7

Table S2: List of plasmids used in this study…………………………………………………………………..9

Figure S1

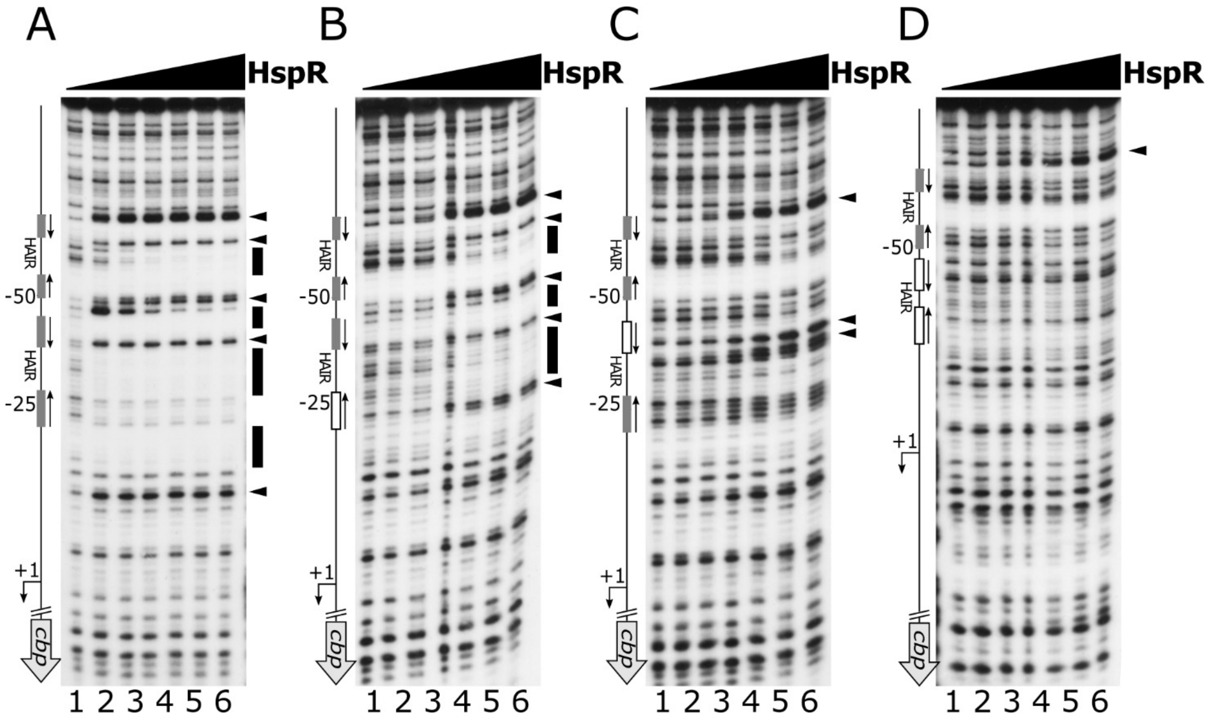

Figure S1. DNase I footprinting assays of HspR on wild type P*cbp* (panel A) and on a set of mutant probes, comprising the mutant of the less conserved HAIR hemisite (panel B), of the more conserved HAIR hemisite (panel C) and the mutant of both HAIR hemisites (panel D). Radiolabelled DNA probes were incubated with increasing concentrations of recombinant HspR protein (0, 22, 45, 90, 180, 360 and 720 nM HspR; lanes 1 to 7, respectively) and subjected to partial DNase I digestion. On the right of each panel, black boxes depict the regions of protection and black arrowheads indicate bands of hypersensitivity to DNase I digestion. On the left side of each panel, a schematic representation of the promoter region, where the bent arrow indicates the transcriptional start site, the vertical open arrow depicts the open reading frame, and the grey boxes alongside converging arrows indicate the HAIR-like sequences; numbers refer to the positions with respect to the transcriptional start site.

Figure S2

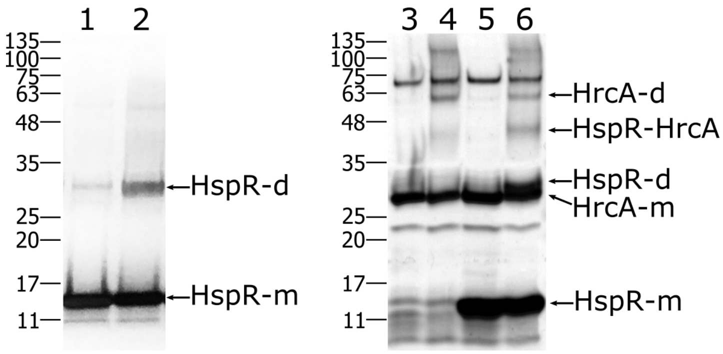

Figure S2. *In vitro* crosslinking assay of HrcA and HspR proteins with formaldehyde. SDS-PAGE of the purified HrcA and HspR proteins not treated (lanes 1, 3, 5) or treated (laned 2, 4 and 6) with 0.01% formaldehyde (Sigma-Aldrich, St Louis, Missouri, USA) in 15 µl of 1X Binding Buffer for 1 hour at 25°C. Chemical crosslinking was halted by adding 5 µl of 5x SDS-PAGE Loading Buffer and boiling each sample at 100°C for 5 minutes. Then, reactions were separated by SDS-PAGE along with a molecular mass ladder (numbers on the left side refers to the molecular weight of protein standards expressed in KDa) and stained with Coomassie Brilliant Blue R-250 (Sigma-Aldrich, St Louis, Missouri, USA). Lanes 1 and 2: HspR alone; lanes 3 and 4: HrcA alone; lanes 5 and 6: HrcA + HspR. The bands corresponding to HspR monomer (HspR-m) and putative dimer (HspR-d), to HrcA monomer (HrcA-m) and putative dimer (HrcA-d), and to the putative HrcA-HspR complex (HspR-HrcA) are indicated on the right.

Figure S3

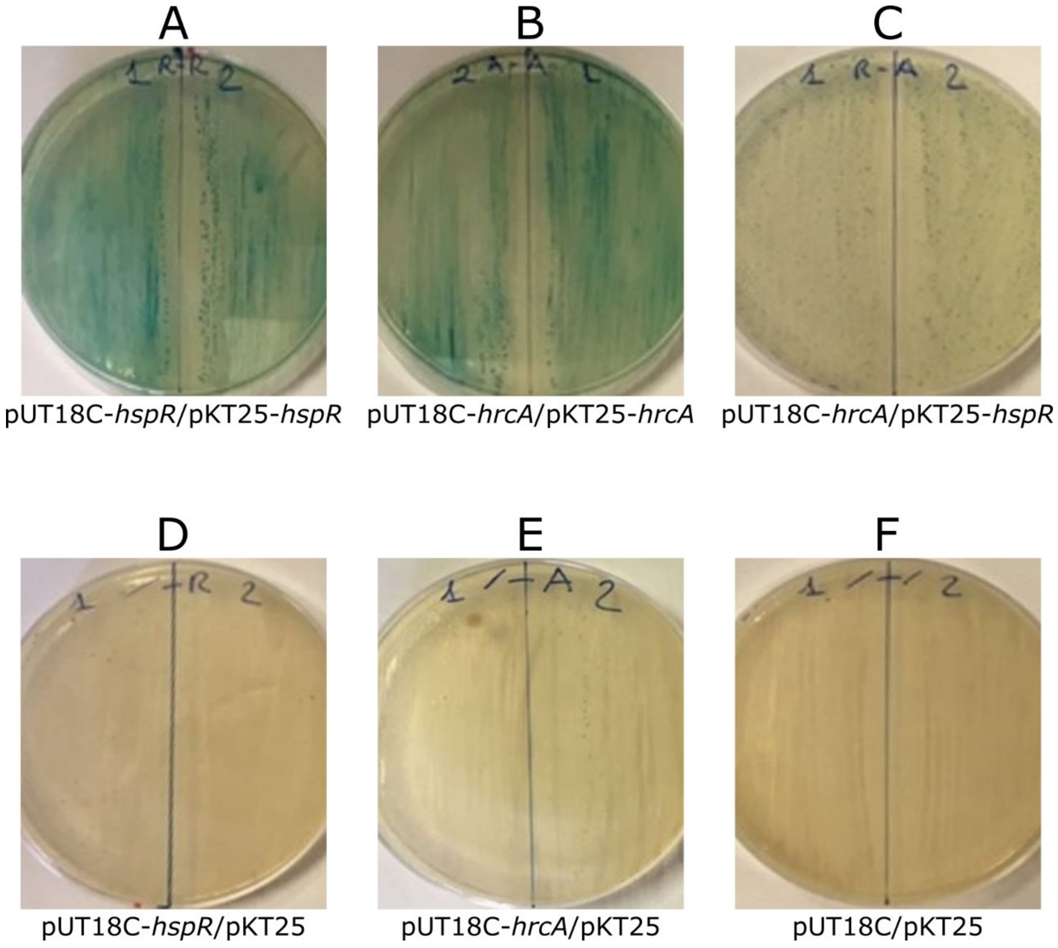

Figure S3. Bacterial Adenylate Cyclase Two-Hybrid System (BACTH) assays [20]. BACTH assay is based on the interaction-mediated reconstitution of the recombinant adenylate cyclase enzyme (CyaA) in *E. coli* (BTH101) strain, which is defective for adenylate cyclase activity. It exploits the fact that the catalytic domain of CyaA from *Bordetella pertussis* consists of two distinct subunits, named T25 and T18. These two subunits are inactive when physically separated, but if they are brought in close proximity to each other, the CyaA activity will be reconstituted. Specifically, the coding sequence of HrcA and HspR were alternatively fused to two complementary T18 and T25 fragments of the CyA enzyme (exploiting the plasmids pUT18C and pKT25, respectively). When the different fusions were expressed in the same cell, upon formation of homo- or hetero-dimers, the CyA catalytic domain was reconstituted and cAMP level increased. Cyclic AMP produced by the reconstituted chimeric enzyme can be indirectly measured by assaying the β-gal enzymatic activities on X-gal chromogenic compound containing plates (development of a blue colour). In detail, *E. coli* BTH101 cells co-transformed with pUT18C-*hspR*/pKT25-*hspR*, pUT18C-*hrcA*/pKT25-*hrcA*, pUT18C-*hrcA*/pKT25-*hspR*, pUT18C-*hspR*/pKT25, pUT18C-*hrcA*/pKT25 or pUT18C/pKT25 (Table S2) were grown on LB agar plates containing 0.5 mM IPTG and 40 μg/ml X-gal (5-Bromo-4-Chloro-3-Indolyl β-D-Galactopyranoside) for 72 h at 25 °C. Results reported in Figure S3 show an evident β-gal activity in the homodimeric interaction, HspR-HspR and HrcA-HrcA (panel A and B). Moreover, the blue colouring developed by cells harbouring pKT25-*hspR* and pUT18C-*hrcA* (panel C), is lower if compared with panel A and B, but well above the background of the three negative controls (panel D, E and F), suggesting the formation of the HspR-HrcA hetero-complex.

Figure S4

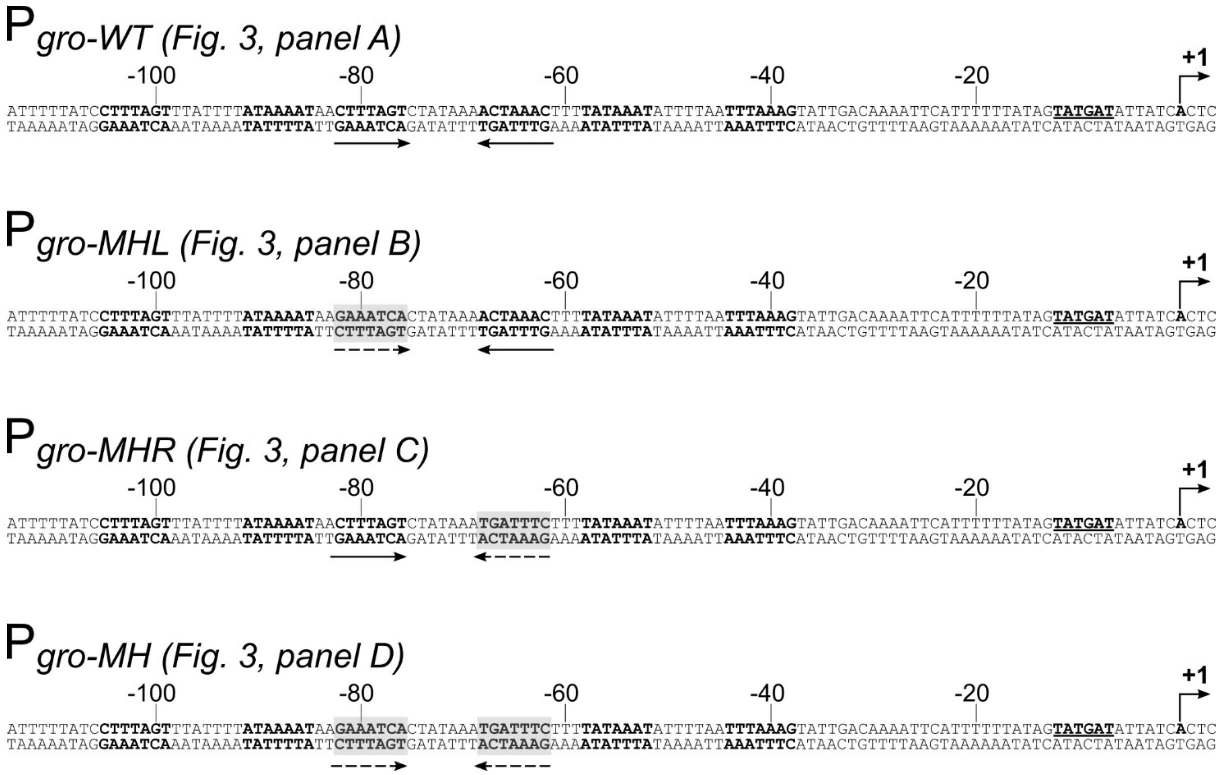

Figure S4. Schematic representation of wild type and three different P*gro* mutant probes used in footprinting experiments reported in Figure 3, in which one (P*gro*-MHL and P*gro*-MHR) or both arms (P*gro*-MH) of the inverted repeat have been mutagenized by base substitution. Only a portion of the full-length probes is represented. For each promoter sequence, the numbers refer to the positions with respect to the transcriptional start site (bent arrow, +1), and the -10 promoter element is in boldface type underlined on the coding DNA strand. The HAIR-like sequences are represented in boldface on both strands. Converging arrows indicate the central high-affinity HAIR-like sequence that was mutated in this set of experiments. In each mutant probe, mutagenized nucleotides are shaded in grey and indicated by a dashed arrow.

Figure S5

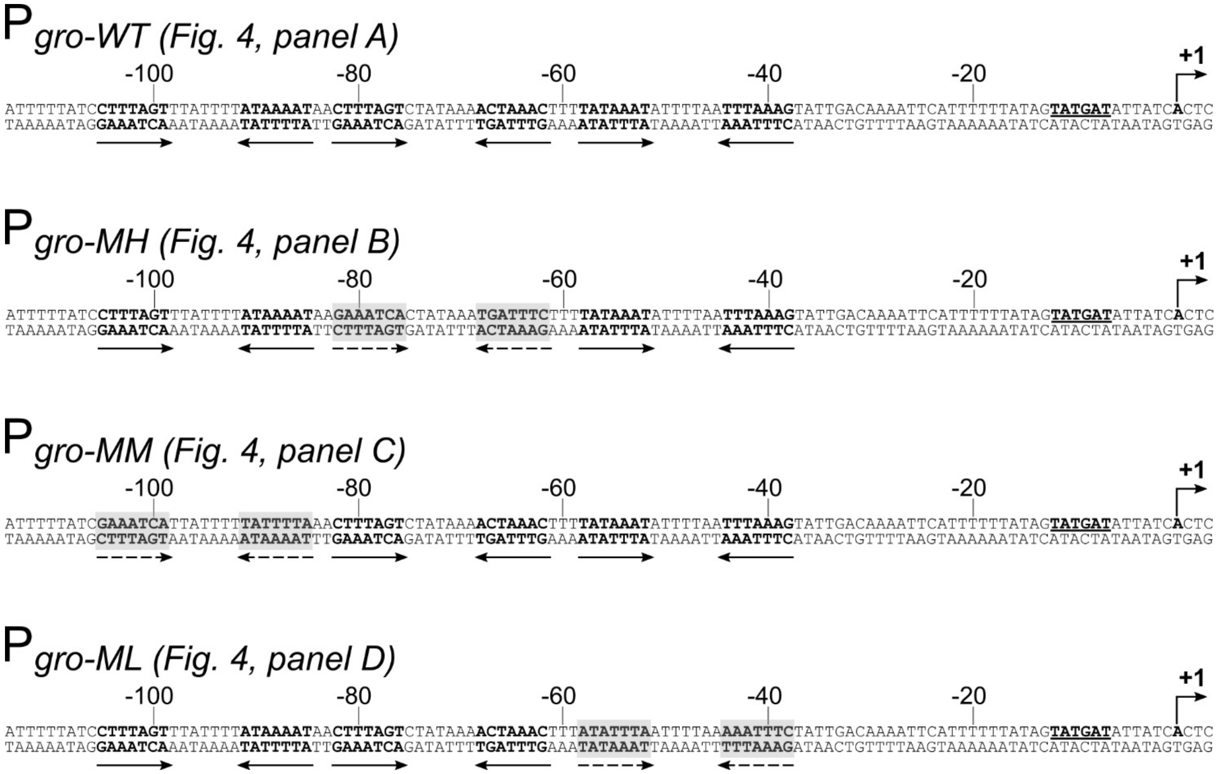

Figure S5. Schematic representation of wild type and three different P*gro* mutant probes used in footprinting experiments reported in Figure 4, in which both arms of the central (P*gro*-MH) or flanking(P*gro*-MM and P*gro*-ML) HAIR-like inverted repeats have been mutagenized by base substitution. Only a portion of the full-length probes is represented. For each promoter sequence, the numbers refer to the positions with respect to the transcriptional start site (bent arrow, +1), and the -10 promoter element is in boldface type underlined on the coding DNA strand. The HAIR-like sequences are represented in boldface on both strands and indicated by converging arrows. In each mutant probe, mutagenized nucleotides are shaded in grey and indicated by dashed arrows.

Table S1 - List of oligonucleotides used in this study.

**Oligonucleotide name**	**Sequence (5′ – 3′)**[a]	**Restriction site**
*CjhrcA*15b-F	CCGCATATGATGAAAAGTCGAGATAAAAAGG	NdeI
*CjhrcA*15b-R	GCGCTCGAGTCACGCCGCCTCCTTTATATATTG	XhoI
*CjhspR*15b-F	CCGCATATGGAACAGCATTATGATGAACC	NdeI
*CjhspR*15b-R	CGGCTCGAGTTATTTTTTCTCATAAAAAATCAAATCAAAGC	XhoI
*Cj*P*cbp*-F	GGATCCCTTGCAGCAAATAAAGCACTTGCTAAAC	BamHI
*Cj*P*cbp*-R	CTCGAGGCTAACTCCAAGAGTTTCGTATAAACTATTC	XhoI
*Cj*P*clp*-F	GGATCCGCATATTATCAGTTAAAAAATCTTGTATATTTGCC	BamHI
*Cj*P*clp*-R	CTCGAGGATATTCAAAGTATAGAAGAATTAAACAAGGC	XhoI
*Cj*P*gro*-F	GGATCCGCACAACAACAAAAGCTACAATGCC	BamHI
*Cj*P*gro*-R	CTCGAGCGCGTTTAACTAGAACACGCTTTCCTAAAGG	XhoI
*Cj*P*hrc*-F	GGATCCGAAGGAAGAAGAATGTATATTTCTATCAATGG	BamHI
*Cj*P*hrc*-R	CTCGAGCCAATAGGTGCATTATCCAAAAGATAAG	XhoI
CjhrcA-F	GCGGGATCCGATGATGAAAAGTCGAGATAAAAAGG	BamHI
CjhrcA-R	CGCGGTACCTCACGCCGCCTCCTTTATATATTG	KpnI
CjhspR-F	GCGGGATCCGATGGAACAGCATTATGATGAACC	BamHI
CjhspR-R	CGCGGTACCTTATTTTTTCTCATAAAAAATCAAATCAAAGC	KpnI
*Cj*P*cbp*WT-F	TAAATAAAACCTTGAGTGATAAAAAATTTATAAAACTTGATTGA<br>CTTAGGCTAAAGTTTATGTTATAATTTAATCCTCTATATAATCAA<br>A	
*Cj*P*cbp*WT-R	TTGATTATATAGAGGATTAAATTATAACATAAACTTTAGCCTAA<br>GTCAATCAAGTTTTATAAATTTTTATCACTCAAGGTTTTATTTAA	
*Cj*P*cbp*MHL-F	TAAATAAAACCTTGAGTGATAAAAAATTTATAAAA**gaactaa**GACTT<br>AGGCTAAAGTTTATGTTATAATTTAATCCTCTATATAATCAAA	
*Cj*P*cbp*MHL-R	TTGATTATATAGAGGATTAAATTATAACATAAACTTTAGCCTAA<br>GTCTTAGTTGTTTTATAAATTTTTATCACTCAAGGTTTTATTTAA	
*Cj*P*cbp*MHR-F	TAAATAAAACCTTGAGTGATAAAAAATTTATAAAACTTGATTGA<br>CTTAG**cgatttc**TTTATGTTATAATTTAATCCTCTATATAATCAAA	
*Cj*P*cbp*MHR-R	TTGATTATATAGAGGATTAAATTATAACATAAAGAAATCGCTA<br>AGTCAATCAAGTTTTATAAATTTTTATCACTCAAGGTTTTATTTA<br>A	
*Cj*P*cbp*MH-F	TAAATAAAACCTTGAGTGATAAAAAATTTATAAAA**gaactaa**GACTT<br>AG**cgatttc**TTTATGTTATAATTTAATCCTCTATATAATCAAA	
*Cj*P*cbp*MH-R	TTGATTATATAGAGGATTAAATTATAACATAAAGAAATCGCTA<br>AGTCTTAGTTGTTTTATAAATTTTTATCACTCAAGGTTTTATTTAA	
*Cj*P*gro*WT-F	AAAATCTTTTTCATTTTTATCCTTTAGTTTATTTTATAAAATAACT<br>TTAGTCTATAAAACTAAACTTTTATAAATATTTTAATTTAAAGTA<br>TTGACAAAA	
*Cj*P*gro*WT-R	CCATCCTTAAAAATATTGATTTTAGCACTCTTTAATTTAGAGTGA<br>TAATATCATACTATAAAAAATGAATTTTGTCAATACTTTAAATT<br>AAAATATTTATAAAAG	
*Cj*P*gro*MHL-F	AAAATCTTTTTCATTTTTATCCTTTAGTTTATTTTATAAAATAA**ga**<br>**aatca**CTATAAAACTAAA**C**TTTTATAAATATTTTAATTTAAAGTAT<br>TGACAAAA	
*Cj*P*gro*MHL-R	CCATCCTTAAAAATATTGATTTTAGCACTCTTTAATTTAGAGTGA<br>TAATATCATACTATAAAAAATGAATTTTGTCAATACTTTAAATT<br>AAAATATTTATAAAAG	

*Cj*P*gro*MHR-F	AAAATCTTTTTCATTTTTATCCTTTAGTTTATTTTATAAAATAACTTTAGTCTATAAA**tgattt**CTTTTATAAATATTTTAATTTAAAGTATTGACAAAA	
*Cj*P*gro*MHR-R	CCATCCTTAAAAATATTGATTTTAGCACTCTTTAATTTAGAGTGATAATATCATACTATAAAAAATGAATTTTGTCAATACTTTAAATTAAAATATTTATAAAAG	
*Cj*P*gro*MH-F	AAAATCTTTTTCATTTTTATCCTTTAGTTTATTTTATAAAATAA**gaaatca**CTATAAA**tgattt**CTTTTATAAATATTTTAATTTAAAGTATTGACAAAA	
*Cj*P*gro*MH-R	CCATCCTTAAAAATATTGATTTTAGCACTCTTTAATTTAGAGTGATAATATCATACTATAAAAAATGAATTTTGTCAATACTTTAAATTAAAATATTTATAAAAG	
*Cj*P*gro*MM-F	AAAATCTTTTTCATTTTTATC**gaaatca**TTATTTT**tatttta**AACTTTAGTCTATAAAACTAAACTTTTATAAATATTTTAATTTAAAGTATTGACAAAA	
*Cj*P*gro*MM-R	CCATCCTTAAAAATATTGATTTTAGCACTCTTTAATTTAGAGTGATAATATCATACTATAAAAAATGAATTTTGTCAATACTTTAAATTAAAATATTTATAAAAG	
*Cj*P*gro*ML-F	AAAATCTTTTTCATTTTTATCCTTTAGTTTATTTTATAAAATAACTTTAGTCTATAAAACTAAACTTT**atattta**ATTTTAA**aaatttc**TATTGACAAA	
*Cj*P*gro*ML-R	CCATCCTTAAAAATATTGATTTTAGCACTCTTTAATTTAGAGTGATAATATCATACTATAAAAAATGAATTTTGTCAATAGAAATTTTTAAAATTAAATATAAAG	

[a] Restriction sites added for cloning purposes are underlined, while mutagenized nucleotides are indicated by lowercase letters.

Table S2 - List of plasmids used in this study.

Plasmid	Description	Reference
pGEM-T-Easy	Cloning vector, Amp^r	Promega
pGEM-T-Easy-*Cj*P*cbp*	pGEM-T-Easy derivative, containing a 285 bp PCR fragment (amplified with oligonucleotides *Cj*P*cbp*-F and *Cj*P*cbp*-R) encompassing the *htrA-cbpA* intergenic region and the 5' parts of the two genes.	This study
pGEM-T-Easy-*Cj*P*clp*	pGEM-T-Easy derivative, containing a 198 bp PCR fragment (amplified with oligonucleotides *Cj*P*clp*-F and *Cj*P*clp*-R) that comprises the P*clp* promoter.	This study
pGEM-T-Easy-*Cj*P*gro*	pGEM-T-Easy derivative, carrying a 261 bp PCR fragment (amplified with oligonucleotides *Cj*P*gro*-F and *Cj*P*gro*-R) encompassing the P*gro* promoter.	This study
pGEM-T-Easy-*Cj*P*hrc*	pGEM-T-Easy derivative, containing a 421 bp PCR fragment (amplified with oligonucleotides *Cj*P*hrc*-F and *Cj*P*hrc*-R) that comprises the P*hrc* promoter.	This study
pGEM-T-Easy-*Cj*P*cbp*WT	pGEM-T-Easy derivative, containing a 89 bp fragment deriving from ssDNA *Cj*P*cbp*WT-F and *Cj*P*cbp*WT-R annealing.	This study
pGEM-T-Easy-*Cj*P*cbp*MHL	pGEM-T-Easy derivative, containing a 89 bp fragment deriving from ssDNA *Cj*P*cbp*MHL-F and *Cj*P*cbp*MHL-R annealing.	This study
pGEM-T-Easy-*Cj*P*cbp*MHR	pGEM-T-Easy derivative, containing a 89 bp fragment deriving from ssDNA *Cj*P*cbp*MHR-F and *Cj*P*cbp*MHR-R annealing.	This study
pGEM-T-Easy-*Cj*P*cbp*MH	pGEM-T-Easy derivative, containing a 89 bp fragment deriving from ssDNA *Cj*P*cbp*MH-F and *Cj*P*cbp*MH-R annealing.	This study
pGEM-T-Easy-*Cj*P*gro*WT	pGEM-T-Easy derivative, containing a 169 bp PCR fragment obtained from the annealing and amplification of the ssDNA *Cj*P*gro*WT-F and *Cj*P*gro*WT-R.	This study
pGEM-T-Easy-*Cj*P*gro*MHL	pGEM-T-Easy derivative, containing a 169 bp PCR fragment obtained from the annealing and amplification of the ssDNA *Cj*P*gro*MHL-F and *Cj*P*gro*MHL-R.	This study
pGEM-T-Easy-*Cj*P*gro*MHR	pGEM-T-Easy derivative, containing a 169 bp PCR fragment obtained from the annealing and amplification of the ssDNA *Cj*P*gro*MHR-F and *Cj*P*gro*MHR-R.	This study
pGEM-T-Easy-*Cj*P*gro*MH	pGEM-T-Easy derivative containing a 169 bp PCR fragment obtained from the annealing and amplification of the ssDNA *Cj*P*gro*MH-F and *Cj*P*gro*MH-R.	This study

pGEM-T-Easy-*Cj*P*gro*MM	pGEM-T-Easy derivative, containing a 169 bp PCR fragment obtained from the annealing and amplification of the ssDNA *Cj*P*gro*MM-F and *Cj*P*gro*MM-R.	This study
pGEM-T-Easy-*Cj*P*gro*ML	pGEM-T-Easy derivative, containing a 169 bp PCR fragment obtained from the annealing and amplification of the ssDNA *Cj*P*gro*ML-F and *Cj*P*gro*ML-R.	This study
pET15b	Expression vector, it allows N-terminal 6X-histidine-tag gene fusion; Amp^r	Novagen
pET15b-*hrcA*	pET15b derivative, containing the *hrcA* coding sequence amplified by PCR with primers *CjhrcA*15b-F and *CjhrcA*15b-R on chromosomal DNA of *C. jejuni*, digested with restriction enzymes NdeI and XhoI and ligated to pET15b.	This study
pET15b-*hspR*	pET15b derivative, containing the *hspR* coding sequence amplified by PCR with primers *CjhspR*15b-F and *CjhspR*15b-R on chromosomal DNA of *C. jejuni*, digested with restriction enzymes NdeI and XhoI and ligated to pET15b.	This study
pGEX-NN	Expression vector, it allows N-terminal GST gene fusion; Amp^r.	Novagen
pGEX-NN-*hrcA*	pGEX-NN derivative, containing the *hrcA* coding sequence excised from pET15b-*hrcA* by NdeI/XhoI digestion.	This study
pGEX-NN-*hspR*	pGEX-NN derivative carrying the *hspR* coding sequence excised from pET15b-*hspR* by NdeI/XhoI digestion.	This study
pUT18C	Expression vector, it allows N-terminal T18 gene fusion; Amp^r.	[20]
pUT18C-*hrcA*	pUT18C derivative, carrying the *hrcA* coding sequence amplified by PCR (with oligonucleotides *CjhrcA*-F and *CjhrcA*-R) and digested with BamHI/KpnI restriction endonucleases.	This study
pUT18C-*hspR*	pUT18C derivative containing the *hspR* coding sequence amplified by PCR with oligonucleotides *CjhspR*-F and *CjhspR*-R, digested with BamHI and KpnI restriction endonucleases.	This study
pKT25	Expression vector, it allows N-terminal T25 gene fusion; Km^r.	[20]
pKT25-*hrcA*	pKT25 derivative, containing the *hrcA* coding sequence, PCR amplified (oligonucleotides *CjhrcA*-F and *CjhrcA*-R), digested BamHI/KpnI restriction endonucleases.	This study
pKT25-*hspR*	pKT25 derivative, carrying the *hspR* coding sequence amplified by PCR (with oligonucleotides *CjhspR*-F and	This study

	CjhspR-R) and digested with BamHI and KpnI restriction enzymes.	
